# *Bombyx mori* histone methyltransferase *BmAsh2* is essential for silkworm piRNA-mediated sex determination

**DOI:** 10.1371/journal.pgen.1007245

**Published:** 2018-02-23

**Authors:** Zhiqian Li, Lang You, Dong Yan, Anthony A. James, Yongping Huang, Anjiang Tan

**Affiliations:** 1 CAS Key Laboratory of Insect Developmental and Evolutionary Biology, CAS Center for Excellence in Molecular Plant Sciences, Shanghai Institute of Plant Physiology and Ecology, Chinese Academy of Sciences, Shanghai, China; 2 Departments of Microbiology & Molecular Genetics and Molecular Biology & Biochemistry, University of California, Irvine, Irvine, California, United States of America; The University of North Carolina at Chapel Hill, UNITED STATES

## Abstract

Sex determination is a hierarchically-regulated process with high diversity in different organisms including insects. The W chromosome-derived *Fem* piRNA has been identified as the primary sex determination factor in the lepidopteran insect, *Bombyx mori*, revealing a distinctive piRNA-mediated sex determination pathway. However, the comprehensive mechanism of silkworm sex determination is still poorly understood. We show here that the silkworm PIWI protein BmSiwi, but not BmAgo3, is essential for silkworm sex determination. CRISPR/Cas9-mediated depletion of *BmSiwi* results in developmental arrest in oogenesis and partial female sexual reversal, while *BmAgo3* depletion only affects oogenesis. We identify three histone methyltransferases (HMTs) that are significantly down-regulated in *BmSiwi* mutant moths. Disruption one of these, *BmAsh2*, causes dysregulation of piRNAs and transposable elements (*TEs*), supporting a role for it in the piRNA signaling pathway. More importantly, we find that *BmAsh2* mutagenesis results in oogenesis arrest and partial female-to-male sexual reversal as well as dysregulation of the sex determination genes, *Bmdsx* and *BmMasc*. Mutagenesis of other two HMTs, *BmSETD2* and *BmEggless*, does not affect piRNA-mediated sex determination. Histological analysis and immunoprecipitation results support a functional interaction between the BmAsh2 and BmSiwi proteins. Our data provide the first evidence that the HMT, BmAsh2, plays key roles in silkworm piRNA-mediated sex determination.

## Author summary

Sex determination is an essential and universal process for metazoan reproduction and development. Insect sex determination is highly diverse, especially for the primary signal and transductory genes. Mechanism of sex determination in the model lepidopteran insect, *Bombyx mori*, is largely unknown, although a piRNA, named *Fem*, has been identified recently as the initial factor. In the current report, we generate somatic mutants for the two silkworm piRNA-bound proteins, *BmSiwi* and *BmAgo3*, and identify that the histone methyltransferase BmAsh2 is involved in silkworm sex determination. Loss of *BmAsh2* function produces a phenocopy of *BmSiwi* mutation and induces partial female-to-male sexual reversal. Importantly, we find the co-localization and protein interaction between BmAsh2 and BmSiwi, further supporting critical roles of *BmAsh2* in the piRNA-mediated sex determination in *B*. *mori*.

## Introduction

Insect sex determination is highly diverse in different species [1,2]. Destiny of the zygote in *Drosophila melanogaster* depends on the number of X chromosome [3–5]. Female flies carry two X chromosomes which activate the transcription of *Sex-lethal* (*Sxl*) and lead to female sexual development, while a single copy of X chromosome in male flies suppresses *Sxl* expression to determine male sexual fate [6,7]. Subsequently, the female-specific Sxl protein regulates splicing of *transformer* (*tra*), which cooperates with the product of the non-sex-specific *transformer 2* (*tra2*) gene to regulate the alternative splicing of *doublesex* (*dsx*) [8,9]. In contrast, the insect WZ sex determination system is found in most lepidopteran insects. For example, in the lepidopteran model insect *Bombyx mori*, females are heterogametic (WZ), while males are homogametic (ZZ) [10,11]. The *B*. *mori* W chromosome exerts a dominant control over sex determination since its presence is sufficient for feminization, and the W chromosome-derived PIWI-interacting RNA (piRNA), named *Feminizer* (*Fem*), has been identified as the primary factor for silkworm sex determination [12]. The *Fem* piRNA is arranged tandemly in the sex determination region of the W chromosome and binds to the PIWI protein BmSiwi to exert its functions [12]. In female silkworms, the *Masculinization* (*BmMasc*) gene is transcribed from the Z chromosome and responsible for both sex determination and dosage compensation. The *Fem* piRNA cleaves the *BmMasc* mRNA in a ping-pong cycler manner to promote the female-specific transcription of *Bmdsx*, resulting in the female fate of animals [10]. Inhibition of *Fem* leads to the production of the male-specific transcript of *Bmdsx* and up-regulates *BmMasc* in female embryos, revealing the critical roles of both *Fem* and *BmMasc* in the silkworm sex determination process, which is distinct from any other species reported [13–15].

The high diversity of sex determination mechanisms indicates that multiple factors may participate in this pathway. Epigenetic modifications are trans-regulators of gene expression that control germline cell imprinting, X chromosome gene inactivation, and gonadogenesis [16]. The histone 3 lysine 9 (H3K9) demethylase, *Jmjd1a*, positively regulates the sex determination gene *Sry* in mice [17]. A lack of *Jmjd1a* causes the H3K9me2 mark to be retained on the *Sry* gene and dysregulation of *Sox9* and *Fox12*, resulting in male-to-female sexual reversal, as demonstrated by the appearance of a uterus in the testis [17–20]. In *B*. *mori*, siRNA-mediated knockdown of the histone methyltransferase (HMT) *DOT1L* (H3K79 methyltransferase) abolishes male-specific expression of *Imp*, an insulin-like growth factor II mRNA-binding protein thought to be a potential regulator of male-specific *dsx* splicing [21]. More recent researches reveal that the prevalent messenger RNA epigenetic modification, N^6^-methyladenosine RNA (m^6^A), controls the alternative splicing of *Sxl* in *Drosophila*, thus functions in the sex determination process [22,23]. These cases indicate that epigenetic modifications, including histone methylation, are involved in sex determination. However, whether histone methylation participates in *B*. *mori* piRNA-mediated sex determination was previously unknown.

The mechanism of silkworm sex determination has long been in mystery until recent identification of the W-derived *Fem* piRNA which functions as the initial signal for silkworm sex determination [12]. Multiply genes that potentially function in the silkworm sex determination pathway have been functional investigated since then [24,25]. However, how does piRNA regulate the downstream sex determination genes remain largely unknown. Here we describe that depletion of the piRNA-bound protein *BmSiwi* causes partial female-to-male sexual reversal, revealing its critical role in silkworm piRNA-mediated sex determination. Furthermore, we find significant down-regulation of three HMTs in *BmSiwi* mutant. Depletion of *BmAsh2*, one of the HMTs, causes partial sexual reversal as well as dysregulation of piRNAs, *TEs*, *Bmdsx* and *BmMasc*. We further demonstrate that there is a functional interaction between the BmSiwi and BmAsh2 proteins. In conclusion, our data provides the first evidence that the HMT *BmAsh2* plays key roles in the silkworm piRNA-mediated sex determination.

## Results

### PIWI proteins express in silkworm gonads predominantly

**Fig 1:**
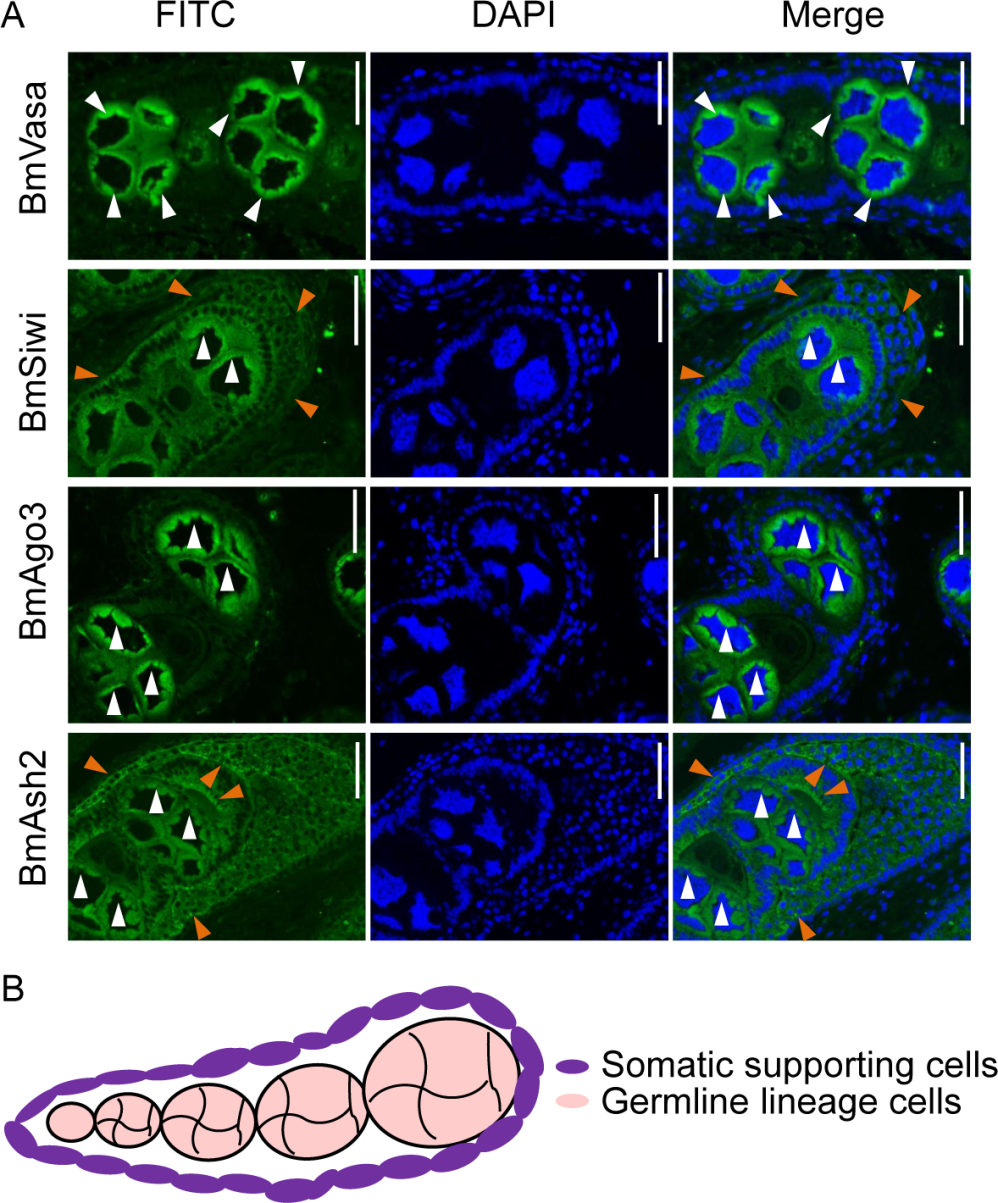
Localization of BmVasa, BmPIWIs (BmSiwi and BmAgo3) and BmAsh2 in silkworm gonads. (A) Proteins localization at larval wandering stag (W) indicated by protein-specific antibodies in silkworm ovaries under immunofluorescence light microscopy. FITC-conjugated Goat-anti-Rabbit secondary antibody was used for fluorescence detection and Hoechst was used to stain nuclei. A BmVasa primary antibody was used to indicate the germline lineage cells. White arrowheads indicate germline lineage cells, and the brown arrowheads indicate somatic supporting cells. Scale bars represent 50 μm. (B) Model for the structure of the silkworm larval ovariole.

Gonad-specific expression of PIWI subfamily proteins (PIWIs) has been identified in the silkworm as well as other organisms [15,26]. In this study, we used qRT-PCR to confirm the predominant expression of two silkworm PIWIs, *BmSiwi* and *BmAgo3*, in gonads at the larval wandering stage (S1A and S1B Fig). The transcript abundance of these two PIWIs was low during the larval stages, increased more than 10-fold after pupation and peaked at the pupal and adult stages in gonads (S1C and S1D Fig). Furthermore, we used immunostaining to investigate the localization of silkworm PIWIs in the gonads at the translational level. Similar to *D*. *melanogaster*, *B*. *mori* ovary possesses several ovarioles which are composed by sequentially developed egg chambers, and serve as an assembly line for oogenesis [27,28]. In order to distinguish the germline and somatic cells in silkworm ovary, we used a primary antibody recognizing BmVasa, which gene has been described as a conserved molecular marker for germline cells in insects, to perform the immunostaining analysis. As the results, distribution of BmVasa and BmAgo3 presented a circular pattern, surrounding the nucleus of germline cells (Fig 1A). In comparison, BmSiwi localized in both the germline cells and the somatic supporting cells which were not stained by the BmVasa antibody (Fig 1A). Localization of silkworm PIWIs was similar to the products of the orthologous genes in *D*. *melanogaster*, suggesting that they may participate in *B*. *mori* piRNA regulation (Fig 1A and 1B) [29,30]. In testis, both BmSiwi and BmAgo3 were detected in the spermatogonium and their distribution completely overlapped with BmVasa (S2 Fig). These results indicated that BmPIWIs may function in gonadogenesis.

### *BmSiwi*, but not *BmAgo3*, is involved in silkworm sex determination

**Fig 2:**
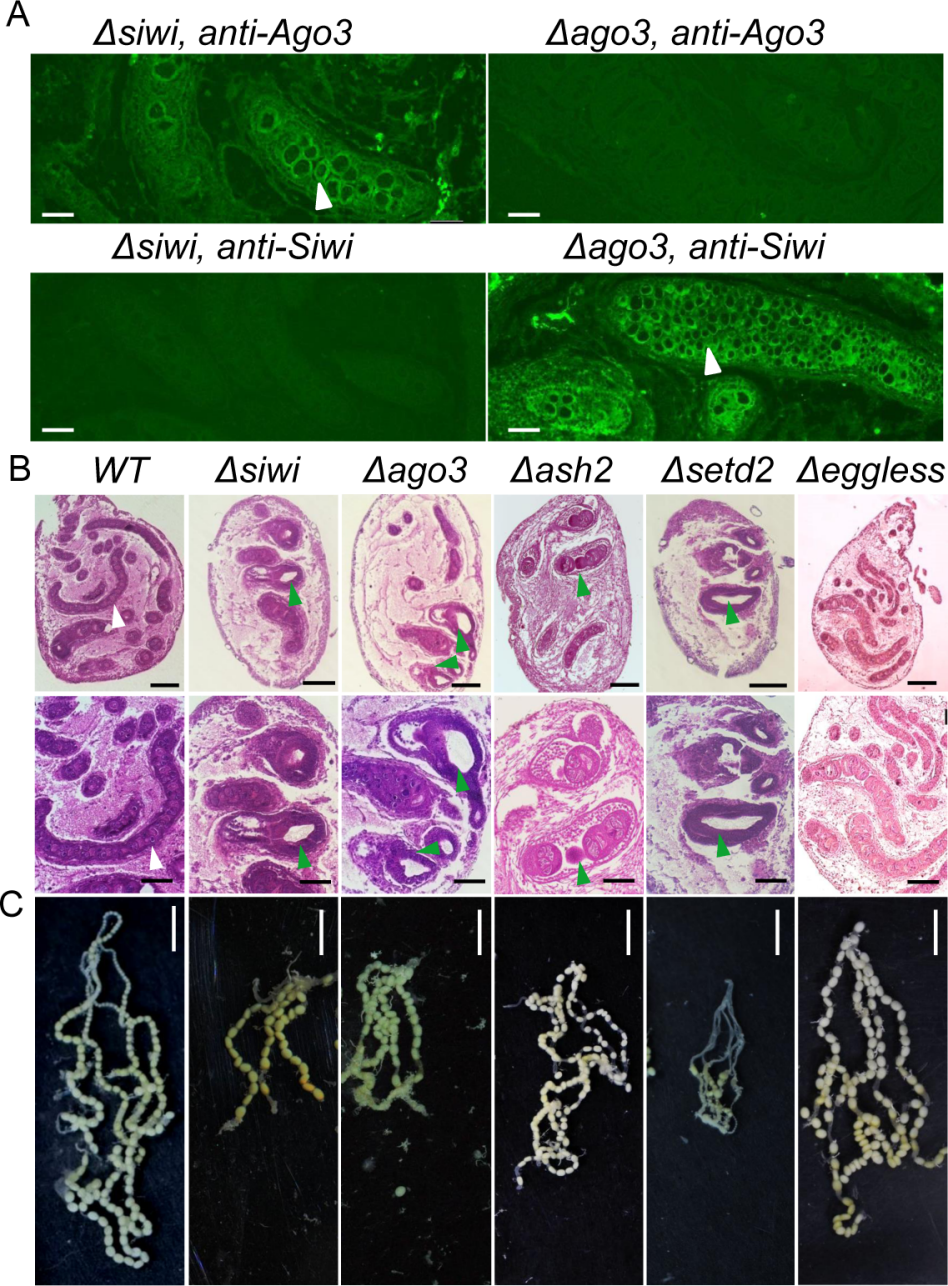
Arrested oogenesis in mutants. (A) Immunohistochemistry in *Δsiwi* and *Δago3* W stage ovaries. The white arrowheads indicate fused egg chambers and the accumulation of germline cells in the ovarioles. Scale bars represent 50 μm. (B) Paraffin-embedded sections of ovaries from *WT*, *Δsiwi*, *Δago3*, *Δash2*, *Δsetd2* and *Δeggless* females at W stage. The lower row showed magnified images (X40). Tissues were stained with hematoxylin-eosin and photographed under a bright field. White arrowheads indicated normal ovariole structures, and green arrowheads indicated the atrophic ovarioles, which were short, vacuolated and contained fused egg chambers in the mutants. Scale bars represent 0.25 mm and 0.125 mm in the upper and lower row respectively. (C) Arrested oogenesis in *Δsiwi*, *Δago3*, *Δash2* and *Δsetd2* mutants. Scale bars represent 0.5 cm.

Using the binary CRISPR/Cas9 system, we established somatic mutant lines for *BmPIWIs* to explore their comprehensive physiological functions (S3A and S3B Fig) [26,31]. Different types of deletions were detected around the target sites in the F_1_ progeny obtained when the *IE1-Cas9* and *U6-sgRNA* transgenic lines were crossed, demonstrating efficient mutagenesis of both genes (S3C and S3D Fig). In addition, the depletion efficiency was further confirmed by histological analysis using corresponding antibodies (Fig 2A).

Compared with wild-type (WT) animals, the larval ovaries from *Δsiwi* and *Δago3* animals were oval-shaped, which was resemble to the WT testis. In details, we observed the development arrested ovarioles were shorter and vacuole filled in both mutants (Fig 2B). As the result, the mature female adults produced few eggs and decreased in fecundity significantly (Fig 2B and 2C). In addition, no clear individual egg chamber was observed in *Δsiwi* and *Δago3* ovarioles since the germline cells divided excessively but differentiated defectively (Fig 2A). However, the testes developed normally in both *Δsiwi* and *Δago3* males, revealing the female-specific function of *BmPIWIs* (S4A Fig). In conclusion, depletion of silkworm *PIWIs* perturbed germline cell development and arrested oogenesis specifically in females.

**Fig 3:**
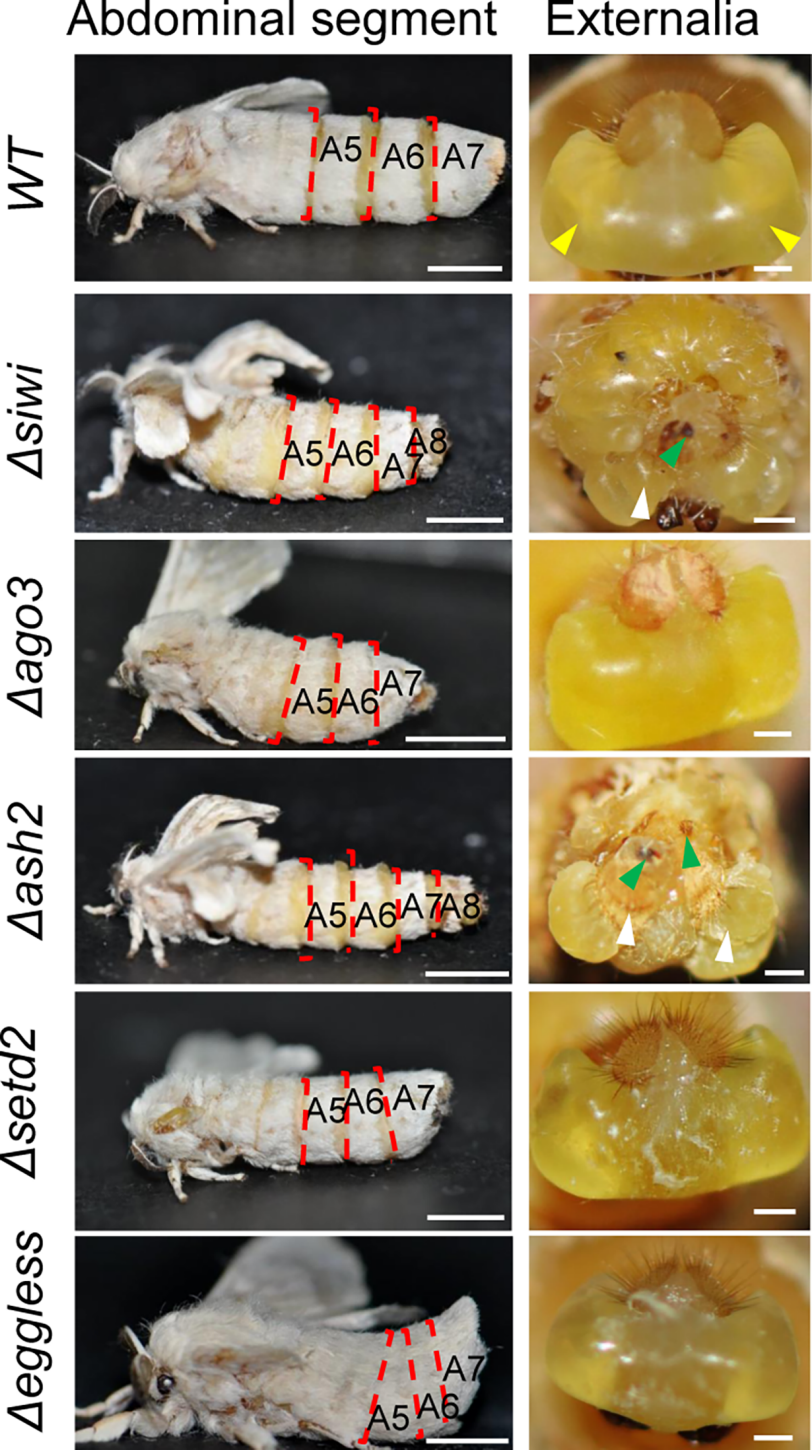
Partial sexual reversal in *Δsiwi* and *Δash2* female animals. Abdominal segments (left column) and female externalias (right column) in *WT*, *Δsiwi*, *Δago3*, *Δash2*, *Δsetd2* and *Δeggless* females were showed. A male-specific 8^th^ abdominal segment in *Δsiwi* and *Δash2* female animals was observed from the lateral view. The WT female animals contain two symmetrical genital papillas as the yellow arrowheads indicated. Both BmSiwi and BmAsh2 female mutants developed clasper-like structures (green arrowheads indicated) and asymmetric differentiated genital papilla (white arrowheads indicated). Scale bars in left and right columns stand for 0.5 cm and 0.5 mm respectively.

Female *Δsiwi* moths developed a male-specific eighth abdominal segment and asymmetrical clasper-like structures on the genital papilla, leading to failure in mating with normal male animals (Fig 3 and S4B Fig). However, neither *Δsiwi* males nor *Δago3* females and males showed developmental defect in abdominal segmentation or the structure of the externalia (Fig 3 and S4C Fig). These partial sexual reversal phenotypes indicated that *BmSiwi* regulates silkworm female sexual dimorphism but *BmAgo3* does not.

**Fig 4:**
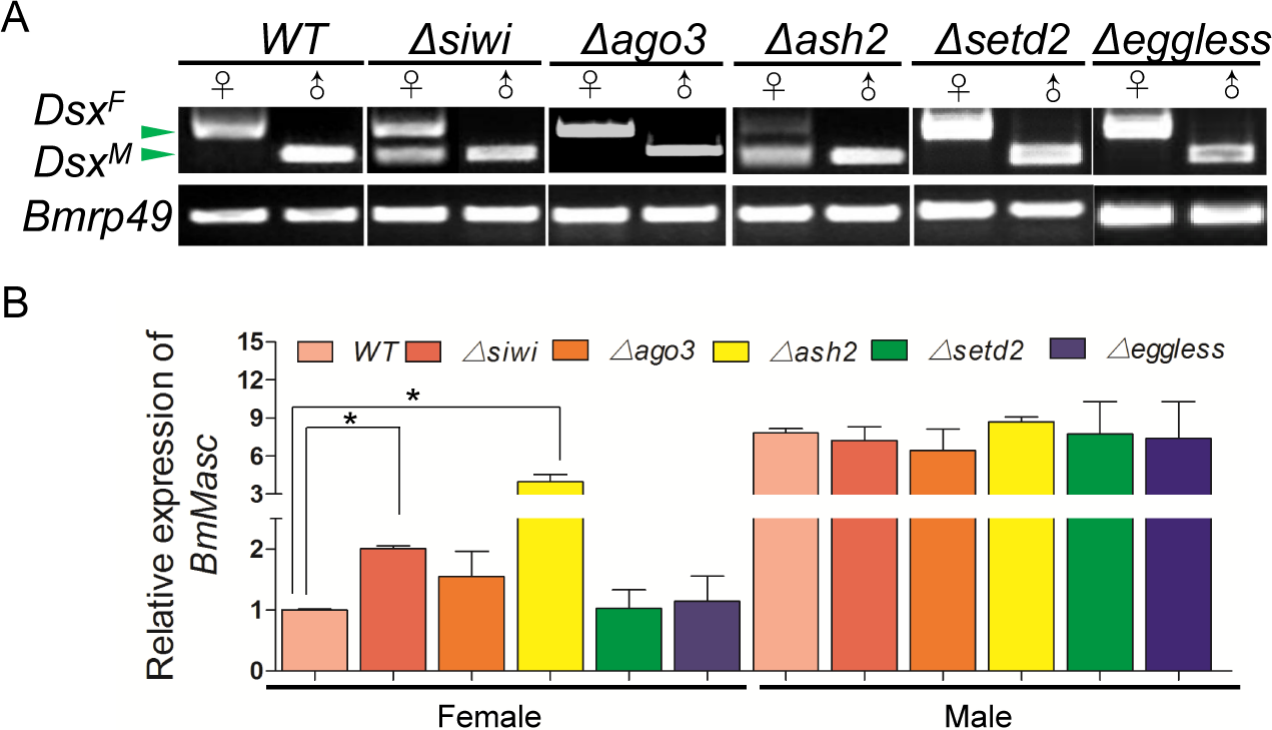
Alternative splicing pattern of *Bmdsx* and relative expression amount of *BmMasc* in WT and mutants. (A) Splicing patterns of the *Bmdsx* gene in mutants. Disruption of *BmSiwi* and *BmAsh2* produced *Bmdsx*^*M*^ (the male-specific transcriptional product of *Bmdsx*) in female animals. (B) Up-regulation of *BmMasc* in *Δsiwi* and *Δash2* females. Silkworm *ribosome protein 49* (*Bmrp49*) was used as the internal reference gene. Three individual replicates were used for qRT-PCR. The error bars represent the mean ± S.E.M and asterisks stand for significance with *p<0*.*05*.

Since the alternative splicing of *Bmdsx* and expression amount of *BmMasc* were the two reporters for masculinization, hence we detected the bands of *Bmdsx* and expression of *BmMasc* in the mutants [12,25,32]. Male-specific splicing production of *Bmdsx* (*Bmdsx*^*M*^) and an increase in *BmMasc* transcript abundance (2.01-fold higher than WT) were detected in *Δsiwi* but not *Δago3* female animals (Fig 4A and 4B), indicating that *BmSiwi* controlled silkworm female sexual dimorphism by regulating *Bmdsx* and *BmMasc*. In addition, no significant change on *Bmdsx* splicing form or *BmMasc* expression was detected in the males of either mutant (Fig 4A and 4B).

### Dysregulation of piRNAs and TEs in *BmSiwi* and *BmAgo3* mutants

RNA-seq analysis was performed using the mixed ovary samples from three individual mutants at the larval wandering stage. In *Δsiwi* females, we identified 1460 differentially-expressed genes (DEGs) in which 1325 genes were down-regulated and 135 genes were up-regulated when compared to WT. In addition, the DEGs were enriched in 268 KEGG terms and 45 GO terms (S5A and S5B Fig). Only 198 DEGs (114 up-regulated and 84 down-regulated) were identified in the *Δago3* females, and these were enriched in 127 KEGG and 36 GO terms (S5A and S5B Fig). Interestingly, the *Δago3* enriched terms completely included in those of *Δsiwi* (S5A and S5B Fig). Two GO items, “reproduction” and “reproduction process”, were identified from both mutants, confirming that BmPIWIs involve in the oogenesis (S5C Fig).

**Fig 5:**
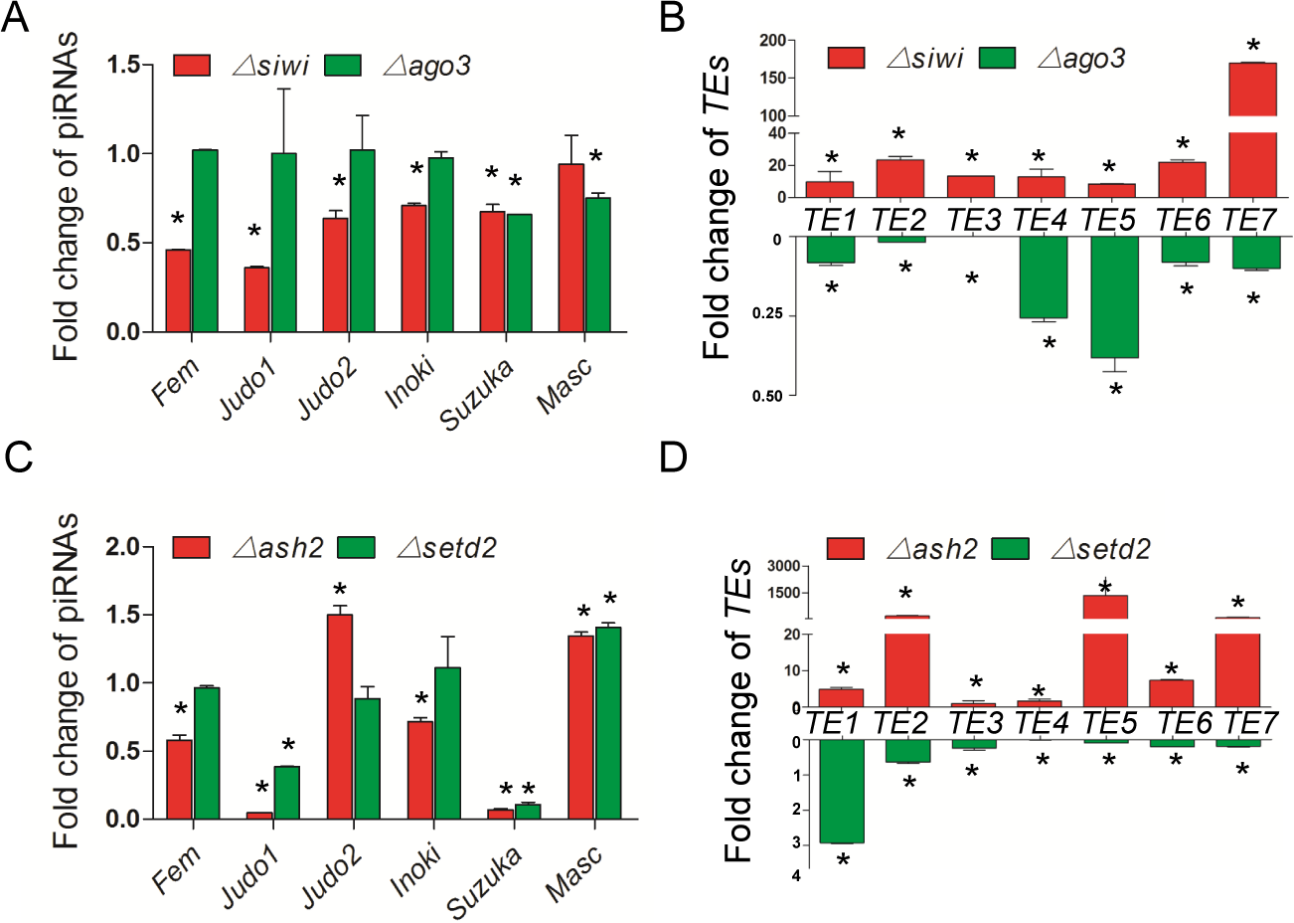
Dysregulation of piRNAs and TEs in mutants. (A and B) Fold change of piRNAs (A) and *TEs* (B) between *Δsiwi* and *Δago3* ovaries, as determined by qRT-PCR. (C and D) Fold change of piRNAs (C) and *TEs* (D) between *Δash2* and *Δsetd2* ovaries. The expression amount was normalized to WT animals. Silkworm *ribosome protein 49* (*Bmrp49*) was used as the internal reference for *TEs*, and the small RNA U6 was used as the internal reference for piRNAs. Three individual replicates were used for qRT-PCR. Error bars represent the mean ± S.E.M. and asterisks stand for significance with *p<0*.*05*.

We also detected significant decrease of piRNA abundance in ovaries of PIWIs female mutants. Comparing to WT females, piRNA abundance decreased to 89.6%, 74.5% and 36.5% in *Δsiwi* females and 95.5%, 85.2% and 66.7% in *Δago3* females for 28-nt, 29-nt and 30-nt piRNAs respectively (S5D Fig). The relative abundance of six known piRNAs, *Fem* (BmSiwi-specific binding piRNA), *Masc* (BmAgo3-specific binding piRNA), *Judo1*, *Judo2*, *Inoki* and *Suzuka* (the latter four of which have no previously-identified binding specificity), were further examined in the two mutants using qRT-PCR. Consistent with previous reports [12], the *Fem* and *Masc* piRNAs were down-regulated in *Δsiwi* and *Δago3* respectively (Fig 5A). Three piRNAs, *Judo1*, *Judo2* and *Inoki*, were down-regulated in *Δsiwi*, but not *Δago3*, supporting the hypothesis that they may be able to bind BmSiwi (Fig 5A). However, the *Suzuka* was down-regulated in both mutants, likely due to a lack of binding specificity between BmSiwi and BmAgo3 (Fig 5A). In addition, qRT-PCR analysis revealed that seven *TEs* were up-regulated in the *Δsiwi* female silkworms but down-regulated in the *Δago3* female animals (Fig 5B). The up-regulation of *TEs* in *Δsiwi* females was expected due to the decrease of its repressor, while this was the first report indicating that disruption of *BmAgo3* induced *TEs* down-regulation. We proposed that this was caused by compensation between the primary and secondary piRNA biosynthesis pathways, although more evidences were needed [10,33]. In conclusion, dysregulation of piRNAs and *TEs* in *Δsiwi* and *Δago3* female animals indicated a conserved function of PIWIs in insects.

### HMT *BmAsh2* is involved in silkworm sex determination

Epigenetic modifications were shown to affect gonadogenesis in *M*. *musculus* and *D*. *melanogaster*, raising the possibility that *B*. *mori* HMTs may participate in piRNA-mediated sex determination [16,34]. Based on the RNA-seq data, qRT-PCR analysis revealed that the transcripts of three HMTs, *BmAsh2*, *BmSETD2* and *BmEggless* decreased in abundance to 67%, 35% and 32% respectively in *Δsiwi* females comparing with WT ones, while no significant difference was found in *Δago3* females (S5E Fig). These three genes showed tissue-specific expression in the gonads and predominantly in the ovaries (S6A–S6C Fig).

We established somatic mutant lines for each HMT using the transgenic CRISPR/Cas9 system to further investigate their physiological roles (S3E–S3G Fig). *Δeggless* animals showed no deleterious phenotype in physiology or sexual development (Figs 2B, 2C and 3). In contrast, *Δash2* and *Δsetd2* animals showed abnormal wing development from pupal stage, resulting in small and curly wings in adults (S7A and S7B Fig). This deleterious phenotype was similar to knock-out phenotypes in *D*. *melanogaster*, in which *Δash2* flies developed absent, small and homeotic wings and *Δsetd2* flies showed blistered wings, indicating a conserved function of *Ash2* and *SETD2* in insect wing morphogenesis [35–37].

*Δash2* and *Δsetd2* females showed defective oogenesis phenotype similar to *Δsiwi* and *Δago3* female moths. Histological analysis revealed that *Δash2* and *Δsetd2* ovaries contained shorter and vacuolated ovarioles (Fig 2B and 2C). However, no defects were observed in the *Δash2* and *Δsetd2* male animals (S4A and S4C Fig). Interestingly, only *Δash2* females showed partial sexual reversal characteristics, such as the appearance of eight abdominal segments and asymmetrically differentiated genital papilla (Fig 3). Furthermore, the *Bmdsx*^*M*^ splicing form and increased *BmMasc* expression were detected in *Δash2* females (Fig 4A and 4B). These results demonstrated that *BmAsh2*, but not *BmSETD2*, was involved in silkworm sex determination.

### BmAsh2 functions as the co-factor of BmSiwi

We further investigated the relationship between HMTs and BmPIWIs because of their similar effects on silkworm female sex determination. We found that piRNAs expressions of *Fem*, *Judo1*, *Inoki* and *Suzuka* were down-regulated in *Δash2* ovaries, consistent with the results found in *Δsiwi* female animals (Fig 5A and 5C). However, in *Δsetd2* ovaries, *Fem*, *Judo2* and *Inoki* levels were comparable to those observed in WT, while *Suzuka* was down-regulated, and this trend was consistent with the results from *Δago3* females (Fig 5A and 5C). The expression of seven *TEs* was up-regulated in *Δash2* females, while all of them, except *TE1*, were down-regulated in *Δsetd2* animals, supporting the hypothesis that the regulation of *BmAsh2* and *BmSETD2* was piRNA-dependent (Fig 5D).

**Fig 6:**
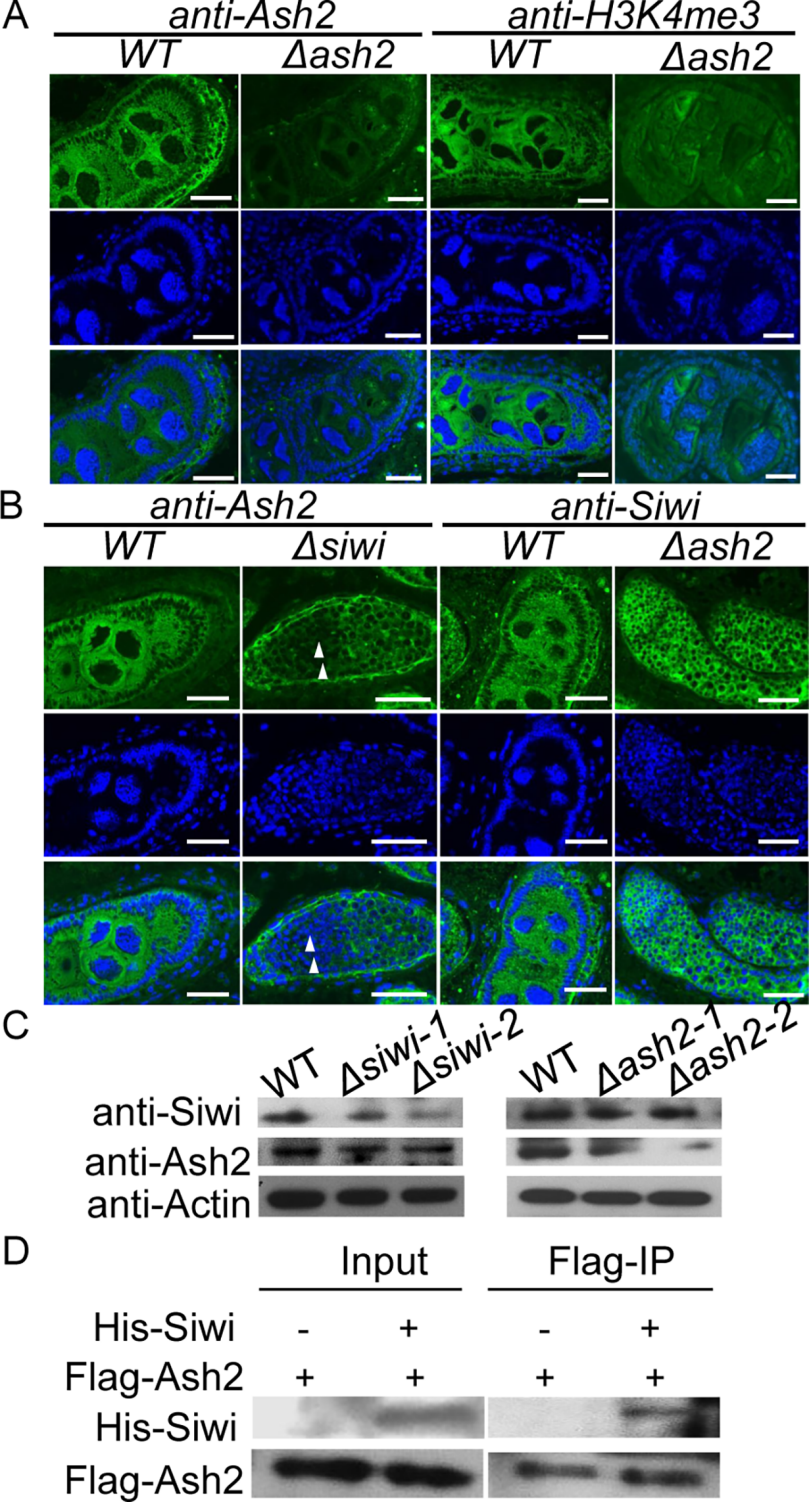
Involvement of HMT *BmAsh2* in piRNA-mediated sex determination. (A) Immunostaining of BmAsh2 and H3K4me3 in WT and *Δash2* ovaries. (B) Immunostaining of BmAsh2 and BmSiwi in *WT*, *Δsiwi* and *Δash2* ovaries. Hoechst was used to stain nuclei in (A) and (B). (C) Western blotting of BmAsh2 and BmSiwi in each mutant detected by anti-BmAsh2 and anti-BmSiwi primary antibody respectively. Actin was used as the internal control. (D) Immunoprecipitation of His-tagged BmSiwi by Flag-tagged BmAsh2 in the silkworm BmN cell line using an anti-Flag primary antibody. Scale bars in (A) and (B) stand for 50 μm.

Since BmAsh2 phenocopied BmSiwi both at the female sexual reversal phenotype and piRNA regulation, we further investigated its localization in silkworm ovary by using immunostaining. BmAsh2 distributed in both the germline and somatic cells in the ovary and accumulated in the spermatogonium of the testis, similar to the localization of BmSiwi (Fig 1A and S2 Fig). Only weak signal of BmAsh2 could be detected in the *Δash2* females, demonstrating that Cas9/sgRNA-mediated mutagenesis was highly efficient (Fig 6A). Since Ash2 is responsible for H3K4me3 modification [38,39], we next examined histone methylation using an anti-H3K4me3 antibody in *Δash2* ovaries and found that the signal decreased significantly, suggesting that H3K4me3-mediated histone methylation was disrupted in *Δash2* animals (Fig 6A). In addition, significant decrease of BmAsh2 protein accumulation was detected in *Δsiwi* ovaries, being consistent to qRT-PCR results (Fig 6B and 6C and S5E Fig). However, both relative mRNA and protein expressions of *BmSiwi* were comparable between *Δash2* and WT ovaries, indicating that *BmAsh2* did not function upstream of BmSiwi in silkworm sex determination pathway (Fig 6B and 6C and S5F Fig).

**Fig 7:**
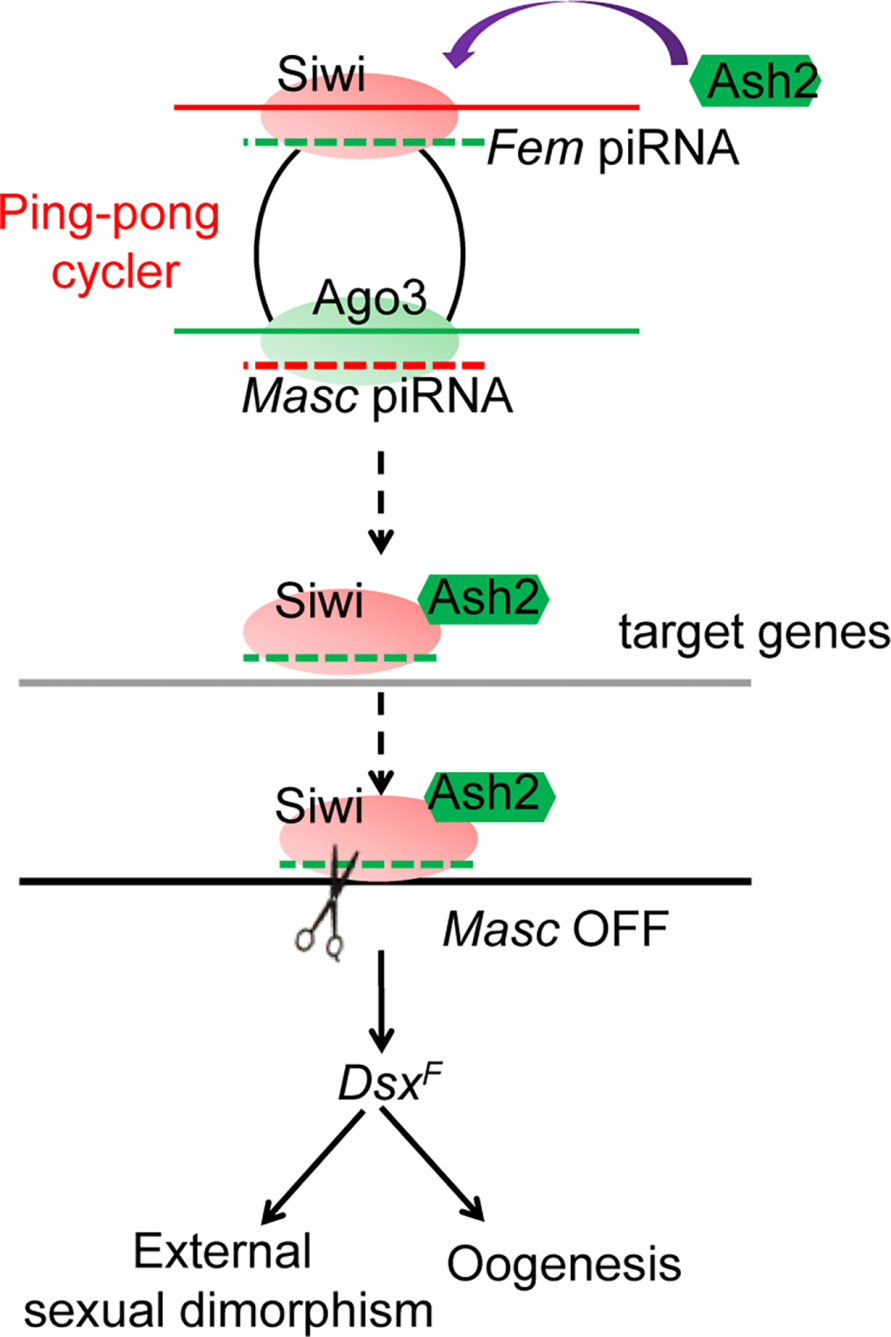
Proposed model for *BmAsh2* involvement in silkworm sex determination. *Fem* piRNA guides the assembly of a transcriptional regulation complex, which possibly includes the *Fem* piRNAs, BmSiwi and BmAsh2 proteins. This complex modifies the transcriptional status of targeting genes. As a result, expression of *BmMasc* is repressed which initiates female-specific splicing of *Bmdsx* and female sex determination, including external sexual dimorphism and oogenesis.

To elucidate the molecular basis of *BmAsh2* involvement in sex determination, we expressed epitope-tagged BmAsh2 and BmSiwi and performed immunoprecipitation in BmN cells, which were derived from silkworm ovaries and exhibit both the primary and secondary piRNA biosynthesis processes. Successful ectopic expression for both proteins were detected in the input samples using anti-His or anti-Flag primary antibodies (Fig 6D). Furthermore, the BmSiwi protein was detected in the Flag immunoprecipitation products, revealing a protein interaction between BmSiwi and BmAsh2. In conclusion, the molecular evidence revealed that *BmAsh2* plays critical roles in BmSiwi- and piRNA-mediated sex determination in *B*. *mori* (Fig 7).

## Discussion

### *BmSiwi* controls female silkworm sex determination

PIWIs belong to the clade of gonadal Argonaute family proteins and silence *TEs* to maintain genomic integrity [15,40,41]. PIWI involvement in gonadal development has been demonstrated by studies showing that depletion of it caused sterility in *Mus musculus*, *D*. *melanogaster* and *Danio rerio* [13,15,42,43]. Absence of the piRNA-bound protein, *Miwi*, *Mili* and *Miwi2*, arrested spermatogenesis at different meiosis stages in mice [13,44,45]. *Drosophila Piwi* depletion caused the accumulation of germline stem cell-like tumors, leading to female infertility [43,46]. Gonadogenesis defect was attributed to DNA damage caused by random *TE* insertion, which disrupted the integrity of the germline stem cell (GSC) genome and homeostasis between GSC self-renewal and differentiation [47,48]. We showed here that a deficiency in *BmSiwi* and *BmAgo3* in the silkworm results in degenerated ovarioles with fused egg chambers and germline cell hyperplasia, revealing the conserved function of PIWIs in gonadogenesis. Since no phenotypic defect was observed in testis development, we concluded that the effect of BmPIWIs on gonadogenesis was female-specific, although high expression of *BmSiwi* and *BmAgo3* was detected in testes.

In addition to its function on oogenesis, *BmSiwi*, but not *BmAgo3*, also was involved in female sex determination. Although *BmSiwi* was reported to function in *Bmdsx* splicing in silkworm embryos [12], there was no previous physiological evidence reported. Here, we found that depletion of *BmSiwi* caused oogenesis arrestment and partial female sexual reversal, including the appearance of additional abdominal segments, asymmetrically differentiated genital papilla and a male-like clasper structure. Furthermore, dysregulation of *BmMasc* expression and splicing of *Bmdsx* further confirmed the function of *BmSiwi* on silkworm sex determination from molecular level. In comparison, no similar phenotype was observed in *Δago3* females, supporting the conclusion that *BmAgo3* does not function in silkworm sex determination. We speculated that the oogenesis arrestment observed in *Δago3* females may be caused by a deficiency in a *dsx*-independent pathway, such as the bone morphogenetic protein (BMP) or epidermal growth factor receptor (EGFR) signaling pathway [49,50]. Thus, the current report provides genetic evidence for the involvement of *BmSiwi* in silkworm sex determination.

### HMT *BmAsh2* is involved in piRNA-mediated sex determination

*Ash2* is part of the SET1/MLL histone methyltransferase complex and is responsible for histone 3 lysine 4 (H3K4) methylation [51–55]. *Drosophila* spermatogenesis is controlled by multiple mechanisms, including epigenetic modifications [56]. In mouse, *TE* expression was repressed by CpG DNA methylation in a Mili-piRNA-dependent manner during sperm development. The repressive histone methylation at H3K9, which was responsible for heterochromatin formation, was active on retrotransposons at the meiotic pachytene stage when DNA methylation was inactive [57]. Expression of a breast tumorigenesis key factor, piRNA-021285, altered the methylation status of a number of related genes [58]. *Drosophila TEs* were silenced by PIWI-piRNA complex-dependent heterochromatin formation along with the silencing signal that spread to its adjacent genes [59]. Furthermore, PIWI-piRNA could recruit an epigenetic factor complex including the heterochromatin protein HP1a and the Su(var)3-9 histone methyltransferase to the target DNA [60]. These data support the conclusion that methylation is critical for gonadogenesis.

We show that the H3K4 HMT *BmAsh2* was functional in piRNA-mediated sex determination in *B*. *mori*. Firstly, loss of *BmAsh2* resulted in phenocopies of the *BmSiwi* mutant in females, which we interpreted to indicate that they function similarly in regulating silkworm sex determination. Furthermore, we detected colocalization of BmSiwi and BmAsh2 in both the germline and somatic cells in silkworm ovary. These two proteins also showed the similar localization at perinucleus in the germline cells, further confirming their important functions in piRNA regulation. More directly, we proved the direct interaction between BmSiwi and BmAsh2 proteins by immunoprecipitation assay. In conclusion, these results support the hypothesis that *BmAsh2* regulates silkworm female sex determination through a piRNA-dependent pathway. Our report provides the first genetic evidence that *BmAsh2* plays critical roles in BmSiwi- and piRNA-mediated silkworm sex determination.

## Materials and methods

### Silkworm strain and cell line

A multivoltine, nondiapausing silkworm strain, Nistari, was used in these experiments. Larvae were reared on fresh mulberry leaves under standard conditions at 25°C [61]. The silkworm ovary-derived cell line BmN used for transfection was cultured at 25°C in TC100 insect medium [31].

### RNA extraction, cDNA synthesis and quantitative real-time PCR (qRT-PCR)

Total RNA was extracted from silkworm ovaries, testes, and other tissues using TRIzol reagent (Invitrogen) according to the manufacturer’s instructions. The isolated RNA was purified with phenol:chloroform and subjected to first-strand cDNA synthesis using the ReverAid First Strand cDNA Synthesis Kit (Vazyme). Relative mRNA amounts were measured using SYBR Green Real-time PCR Master Mix (Toyobo) according to a previously described method [31]. The qRT-PCR primers used here were as following: *BmSiwiRTF*: 5’-ATCACCCCAGAAAGACAACG-3’, *BmSiwiRTR*: 5’-GCACAGTATCAGGGCAGGAT-3’, *BmAgo3RTF*: 5’-GAGCAGTGCACAAAGCGATA-3’, and *BmAgo3RTR*: 5’-GGCACACCTGTTTCACCTTT-3’. As an internal control for qRT-PCR, we used a primer set that amplified a 136-bp PCR product of *B*. *mori ribosomal protein 49* (*Bmrp49*) [31]. Three independent biological replicates were used for qRT-PCR, and other primers are listed in S1 Table. PiRNA sequences were found by referring to Kawaoka *et al*. [11], and the relative expression was measured using the stem-loop method [62].

### CRISPR/Cas9-mediated construction of mutants

A binary transgenic CRISPR/Cas9 system was used to construct silkworm mutants as described in Li et al. [31]. Six plasmids were constructed: the first, *pBac[IE1-DsRed-IE1-Cas9]* (*IE1-Cas9*), expresses the Cas9 protein constitutively driven by the baculovirus immediate-early gene IE1 promoter; and the other five, *U6-BmSiwi sgRNA* (*pBac[A3-EGFP-U6-BmSiwi sgRNA]*), *U6-BmAgo3 sgRNA*, *U6-BmSETD2 sgRNA*, *U6-BmAsh2 sgRNA* and *U6-BmEggless sgRNA*, express small guide RNAs (sgRNAs) targeted to *BmSiwi* (5’- CCTGAGTTGATATATCTAGTGCC-3’), *BmAgo3* (5’-GGAGTGAGTATAGGCGGTAGAGG-3’), *BmSETD2* (5’- CCATTAGCTAGTCCAGGTCTGCC-3’), *BmAsh2* (5’-GGCAACGTGAAGGGCAGGCAAGG-3’) and *BmEggless* (5’- GGAGGCGGCGCAGCTCCGCGCGG-5’), respectively, under the control of the silkworm U6 small nuclear RNA promoter.

The plasmids were injected into preblastoderm embryos with a mixture of helper plasmids, *piggyBac* transposon mRNA and transgenic vectors. G_0_ animals were incubated at 25°C for 10–12 d until hatching, fed with fresh mulberry leaves, sib-mated or back-crossed with WT moths, and screened at late G_1_ embryos under a fluorescence microscope (Nikon, AZ100). Crossing the *IE1-Cas9* and *U6-sgRNA* transgenic silkworms generates the gene-specific mutants used for the following experiments.

### High-throughput sequencing analysis of mRNA and piRNA

Total RNA from the ovary of wandering stage (when the ovary undergoes maturation) animals was extracted from three individual animals of *Δsiwi*, *Δago3* and *WT* and mixed together. For mRNA sequencing, mRNA was enriched with Sera-mag Magnetic Oligo(dT) Beads (Illumina), fragmented to 200 nt in average, and used for cDNA synthesis. After that, the cDNA was sent to purification, end repair, nucleotide A and adapters addition (Illumina). Subsequently, the modified RNA was amplified with PE 1.0 and PE 2.0 PCR primers for 15 rounds and sequenced on an Illumina HiSeq 2000 platform (Shanghai OE BIOTECH CO., LTD). Sequenced raw data was qualified, filtered, and mapped to the reference silkworm genome database (http://silkworm.genomics.org.cn/) using tophat/bowtie2. Unigene abundance was measured by fragment per kilobase of exon per million fragments mapped (FPKM) and used for subsequent annotation.

RNA samples extracted from the ovary were also used for piRNA sequencing. Ten micrograms RNA was separated using 15% denaturing polyacrylamide gels and the small RNAs in length from 18 to 30 nt were used to construct library. Subsequently, small RNAs were sent to adaptors ligation at both the ends, cDNA synthesis and amplification were performed by using small RNA Cloning Kit (Takara). After sequencing with illumine HiSeq 2500 platform, the generated reads were filtered and small RNA reads from 24 to 30 nt in length were selected for mapping to the silkworm genome (http://silkworm.genomics.org.cn/silkdb/#), 121 annotated transposons and 1668 ReAS clones to identify the piRNAs as reported previously [63].

### Paraffin embedding and hematoxylin-eosin staining

Silkworm ovaries and testes dissected from WT, *Δsiwi*, *Δago3*, *Δash2*, *Δsetd2* and *Δeggless* animals at larval wandering stage were prefixed with Qurnah’s fixative [31]. Cross sections of 5 μm were cut with a Leica RM2235 microtome and used for staining. Sections were hydrated and stained with hematoxylin solution for 2 min, washed with running tap water for 5 min, stained with eosin solution for 2 min and dehydrated with 95% and 100% ethanol for 2 min each [64]. The stained tissues were analyzed and photographed under a microscope (Olympus BX51, Japan).

### Silkworm gonad immunohistochemistry

Paraffin-embedded sections were rehydrated and subjected to antigen retrieval with 0.1% trisodium citrate containing 0.1% Triton X-100 for 10 min at room temperature. The samples were washed with phosphate buffered saline (PBS) once and blocked with 1% bovine serum albumin (BSA) for 1 hour at room temperature. The silkworm gonads were incubated with Rabbit anti-BmVasa (1:200, Youke Biotech, indicating the germline lineage cells) [65], anti-BmSiwi (1:200, Youke Biotech), anti-BmAgo3 (1:200, Youke Biotech), anti-BmAsh2 (1:200, Youke Biotech) and anti-H3K4me3 (1:200, ABclonal) primary antibodies for 48 hours at 4°C. Samples were washed with PBS twice and treated with a FITC-conjugated Goat-anti-Rabbit secondary antibody (diluted 1:100 with 1% BSA, YEASEN) for 2 hours. Nuclei were stained with Hoechst (Beyotime) for 10 min at room temperature. After staining, samples were washed three times with PBS and analyzed with a fluorescence microscope (Olympus, BX53).

### Immunoprecipitation

Flag-tagged *BmAsh2* and His-tagged *BmSiwi* coding sequences were cloned into the *pIZT/V5-His A* insect expression plasmid under the control of an optimized baculovirus immediate-early gene promoter IE2 (OpIE2). The plasmids were transfected into the silkworm ovary-derived cell line BmN using Effectene transfection reagent (Qiagen) according to the manufacturer’s instructions. Three days after transfection, crude proteins were extracted and used for immunoprecipitation with a mouse monoclonal anti-Flag M2 antibody (1:1000, Sigma) according to Song et al. [66]. BmSiwi was detected using a Mouse anti-His (1:1000, Youke Biotech) primary antibody.

### Statistical analysis of data

All data were analyzed using GraphPad Prism (version 5.01) with two-way ANOVA and the Dunnett’s tests. All error bars were the means ± S.E.M. *p<0*.*05* was used to determine significance in all cases.

## Supporting information

S1 Fig Spatial and temporal expression patterns of BmPIWIs in silkworm gonads. (A and B) Expression profile of *BmSiwi* (A) and *BmAgo3* (B) in six major tissues of silkworm at larval wandering stage (W). Epi: epidermis, MG: midgut, FB: fat body, SG: silk gland, Ov: ovary, Te: testis. (C and D) Temporal expression profile of *BmSiwi* (C) and *BmAgo3* (D) in gonads from day one of the fifth instar larvae (L5D1) to adult (A). PP1: day one of pre-pupae, P1: day one pupae. The relative transcription levels of *PIWIs* were determined by qRT-PCR and normalized to the internal reference gene *ribosome protein 49* (*Bmrp49*). Three individual biological replicates were used for qRT-PCR. The data shown are the mean ± S.E.M.
(TIF)

S2 Fig Distribution of BmVasa, BmSiwi, BmAgo3 and BmAsh2 in the spermatogonium of the silkworm testis at larval wandering stage. The corresponding localizations in silkworm testes were detected using protein-specific antibodies at larval wandering stage. The white arrowheads indicate spermatogonium cells. Scale bars represent 100 μm.
(TIF)

S3 Fig Construction of somatic mutants using the binary CRISPR/Cas9 system. (A) Location of *BmSiwi* and *BmAgo3* on silkworm chromosome 12 and 3, respectively. The gene sequences are represented by blue bars, and the sgRNA targeting sequences are listed below. (B) Schematic diagrams of plasmids used for Cas9 protein and sgRNA expression. The plasmid *IE1-Cas9* was used to express Cas9 driven by the ubiquitous baculovirus immediate-early gene IE1 promoter, and sgRNAs were driven by the U6 small nuclear RNA promoter. Purple arrows: promoters, black arrows: right and left inverted terminal repeats of the *piggyBac* transposon, yellow box: Cas9 protein coding sequence or sgRNAs, red or green box: selection markers expressing DsRed or enhanced green fluorescence protein (EGFP), gray box: polyadenylation sequence of SV40 for expressing Cas9 protein or polyT for sgRNAs. (C-G) Various types of deletions (C for *Δsiwi*, D for *Δago3*, E for *Δash2*, F for *Δsetd2* and G for *Δeggless*) in the heterozygous offspring after crossing the sgRNA transgenic lines with *IE1-Cas9* transgenic animals. Red letters indicate the target sequences, and green letters are PAM (protospacer adjacent motifs) sequences.
(TIF)

S4 Fig Phenotype of testis and male externalias in WT and mutant. (A) Paraffin-embedded sections of WT and mutant testes. The scale bars represent 0.5 mm in the upper row and 0.25 mm in the lower row. The lower row shows the magnification (X40) of the images in the upper row (X20). (B) Abdominal segment from the lateral view in *WT* male. (C) Structure of externalias in WT, *Δsiwi*, *Δago3*, *Δash2*, *Δsetd2* and *Δeggless* males. Claspers are indicated by white arrowheads. Scale bars stand for 0.5 cm and 0.5 mm in (B) and (C) respectively.
(TIF)

S5 Fig Summary of the RNA-seq results from *WT*, *Δsiwi* and *Δago3* ovaries. (A and B) Venn diagrams of enriched KEGG and GO terms between *Δsiwi* and *Δago3* ovaries. (C) The top significantly enriched GO terms in *Δsiwi* and *Δago3* ovaries. The green arrowheads indicate two processes related to oogenesis. (D) Abundance of small RNAs ranging from 24 to 30 nt. Arrows indicate the decrease in piRNA abundance. (E) Fold changes of *BmAsh2*, *BmSETD2* and *BmEggless* in *Δsiwi* and *Δago3* females normalized to WT. Asterisks stand for significance with *p<0*.*05*. (F) Relative transcript abundance of *BmSiwi* in *Δash2* ovaries. The silkworm *ribosome protein 49* (*Bmrp49*) ortholog was used as the internal reference gene in (E) and (F). Three individual replicates were used for qRT-PCR, and the error bars represent the mean ± S.E.M.
(TIF)

S6 Fig Spatial expression pattern of three HMTs in silkworm larval tissues. Six major tissues, including Epi, MG, FB, MSG, Ov and Te, were sampled from W larvae and used for investigation. Three individuals were used for qRT-PCR. The error bars represent the mean ± S.E.M.
(TIF)

S7 Fig Defects in wing morphogenesis caused by depletion of *BmAsh2* and *BmSETD2*. (A) Female (upper) and male (lower) pupae at day 5 after puparium. White arrowheads indicate abnormal wing discs in pupae of *Δash2* and *Δsetd2* animals. (B) Abnormal wings from WT adult, *Δash2* and *Δsetd2* day nine pupae. The upper are fore wings and lower are hind wings. Scale bars represent 0.5 cm.
(TIF)

S1 Table Primers used in this work. (DOCX)
