# Supplementary figures and images for: Bombyx mori histone methyltransferase BmAsh2 is essential for silkworm piRNA-mediated sex determination
Source: PLoS Genet. 2018 Feb 23;14(2):e1007245. doi: 10.1371/journal.pgen.1007245 (PMC5841826; doi:10.1371/journal.pgen.1007245)

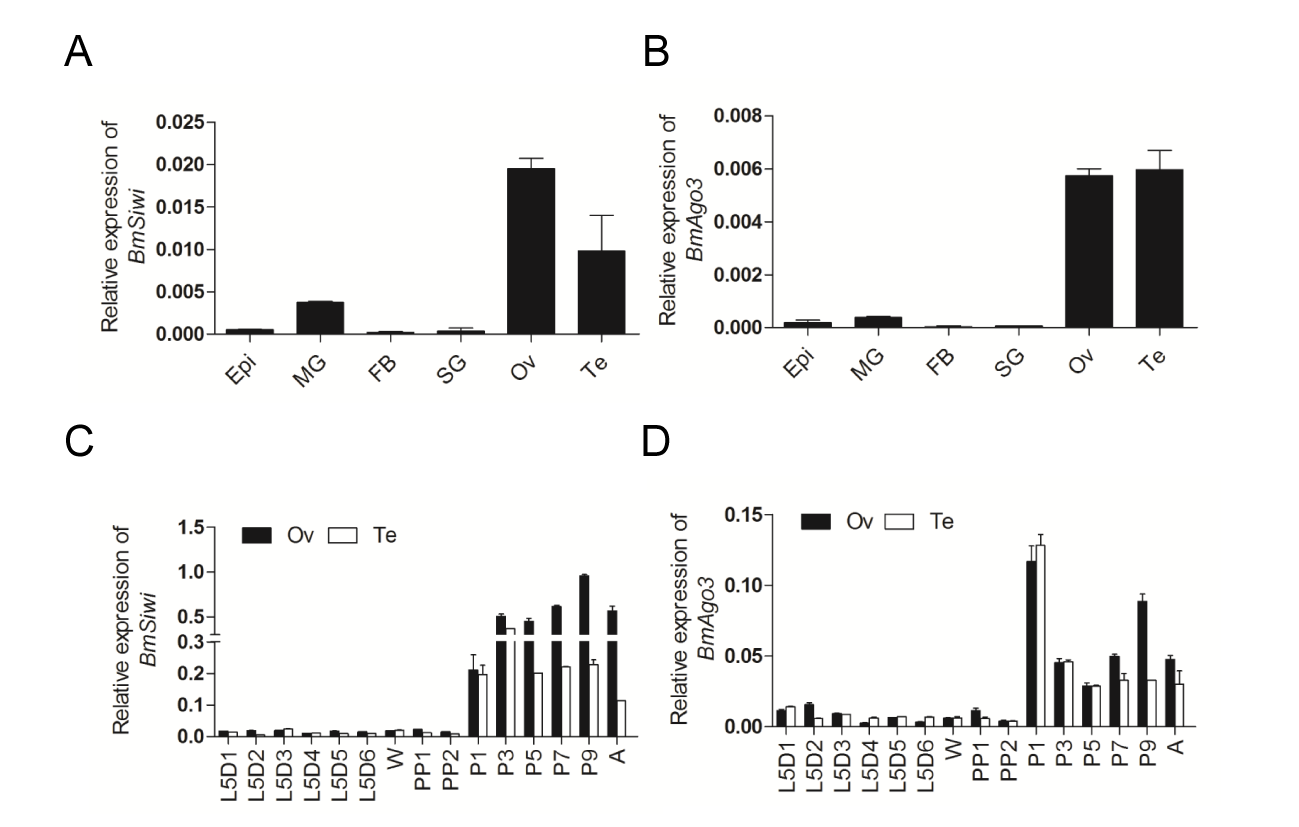

Supplement: S1 Fig — (A and B) Expression profile of BmSiwi (A) and BmAgo3 (B) in six major tissues of silkworm at larval wandering stage (W). Epi: epidermis, MG: midgut, FB: fat body, SG: silk gland, Ov: ovary, Te: testis. (C and D) Temporal expression profile of BmSiwi (C) and BmAgo3 (D) in gonads from day one of the fifth instar larvae (L5D1) to adult (A). PP1: day one of pre-pupae, P1: day one pupae. The relative transcription levels of PIWIs were determined by qRT-PCR and normalized to the internal reference gene ribosome protein 49 (Bmrp49). Three individual biological replicates were used for qRT-PCR. The data shown are the mean ± S.E.M. (TIF) [file pgen.1007245.s001.tif]

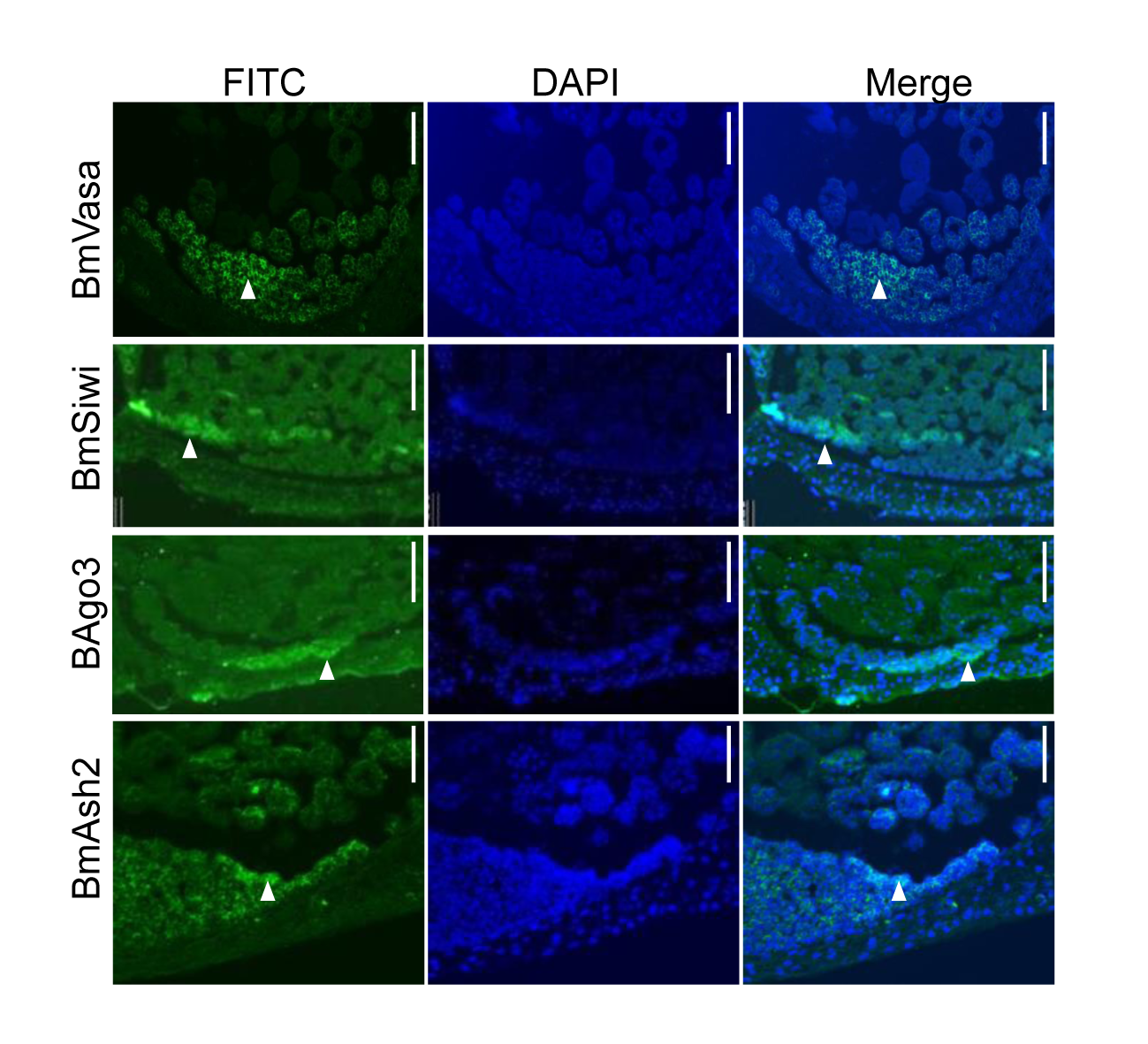

Supplement: S2 Fig — The corresponding localizations in silkworm testes were detected using protein-specific antibodies at larval wandering stage. The white arrowheads indicate spermatogonium cells. Scale bars represent 100 μm. (TIF) [file pgen.1007245.s002.tif]

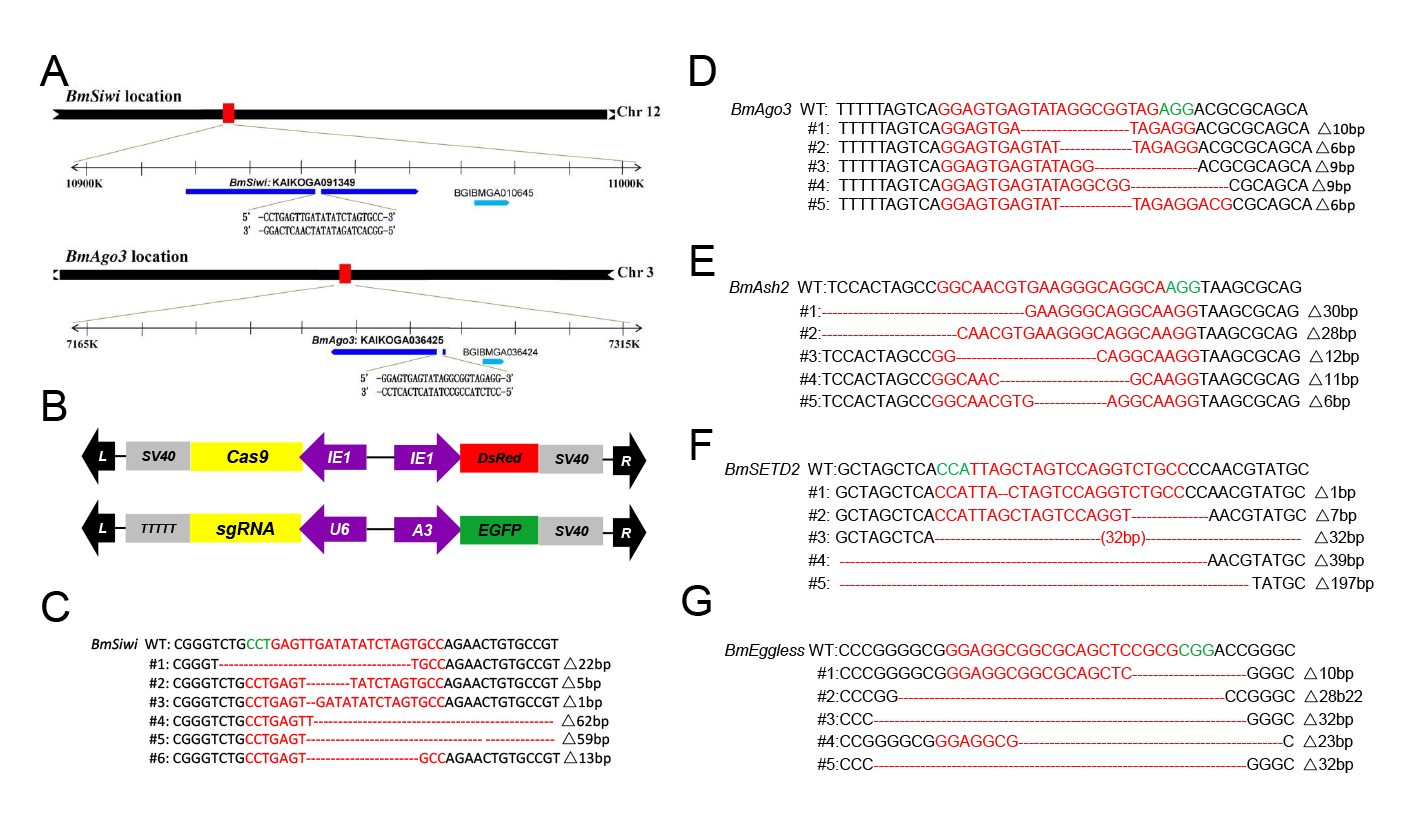

Supplement: S3 Fig — (A) Location of BmSiwi and BmAgo3 on silkworm chromosome 12 and 3, respectively. The gene sequences are represented by blue bars, and the sgRNA targeting sequences are listed below. (B) Schematic diagrams of plasmids used for Cas9 protein and sgRNA expression. The plasmid IE1-Cas9 was used to express Cas9 driven by the ubiquitous baculovirus immediate-early gene IE1 promoter, and sgRNAs were driven by the U6 small nuclear RNA promoter. Purple arrows: promoters, black arrows: right and left inverted terminal repeats of the piggyBac transposon, yellow box: Cas9 protein coding sequence or sgRNAs, red or green box: selection markers expressing DsRed or enhanced green fluorescence protein (EGFP), gray box: polyadenylation sequence of SV40 for expressing Cas9 protein or polyT for sgRNAs. (C-G) Various types of deletions (C for Δsiwi, D for Δago3, E for Δash2, F for Δsetd2 and G for Δeggless) in the heterozygous offspring after crossing the sgRNA transgenic lines with IE1-Cas9 transgenic animals. Red letters indicate the target sequences, and green letters are PAM (protospacer adjacent motifs) sequences. (TIF) [file pgen.1007245.s003.tif]

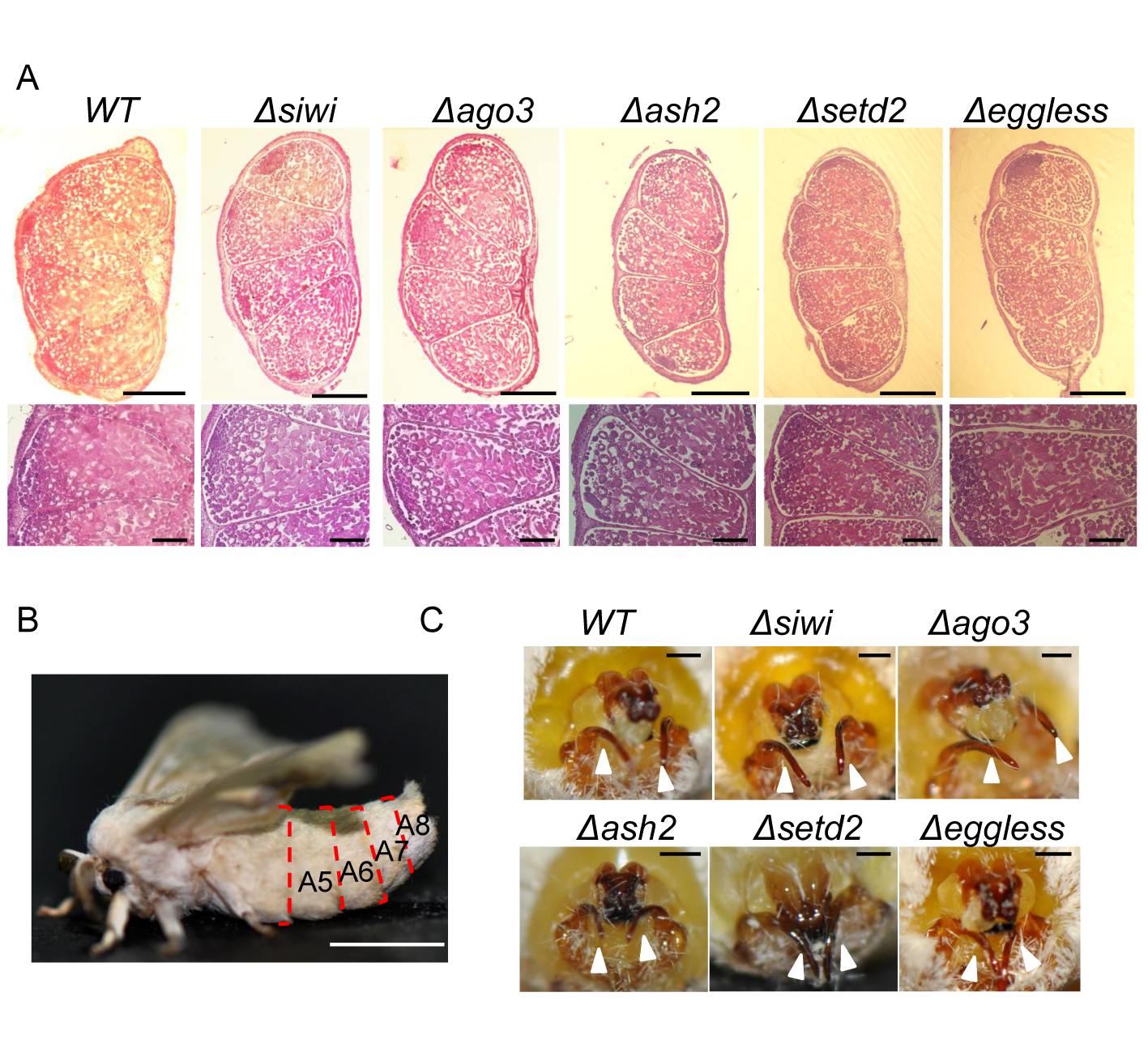

Supplement: S4 Fig — (A) Paraffin-embedded sections of WT and mutant testes. The scale bars represent 0.5 mm in the upper row and 0.25 mm in the lower row. The lower row shows the magnification (X40) of the images in the upper row (X20). (B) Abdominal segment from the lateral view in WT male. (C) Structure of externalias in WT, Δsiwi, Δago3, Δash2, Δsetd2 and Δeggless males. Claspers are indicated by white arrowheads. Scale bars stand for 0.5 cm and 0.5 mm in (B) and (C) respectively. (TIF) [file pgen.1007245.s004.tif]

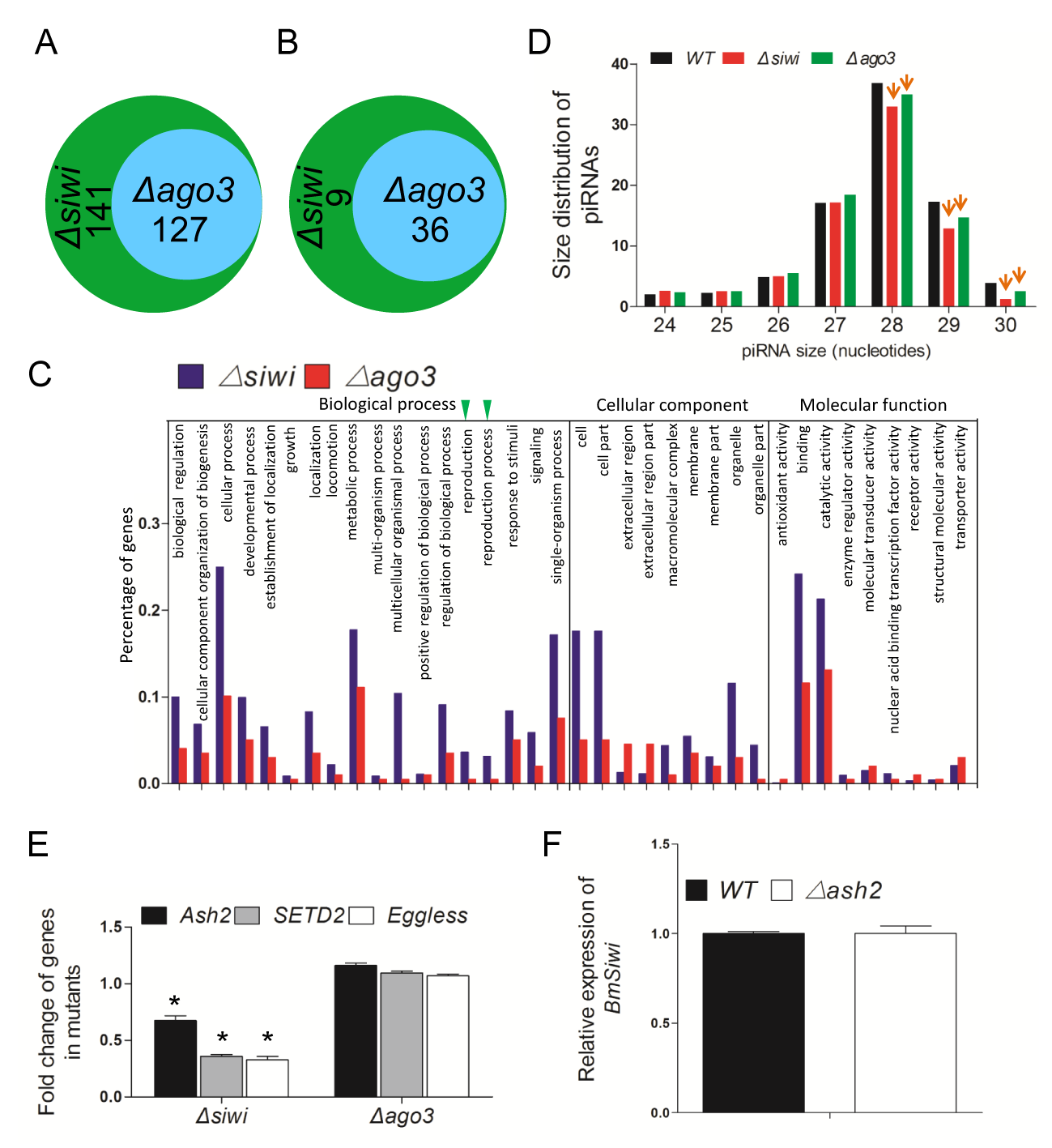

Supplement: S5 Fig — (A and B) Venn diagrams of enriched KEGG and GO terms between Δsiwi and Δago3 ovaries. (C) The top significantly enriched GO terms in Δsiwi and Δago3 ovaries. The green arrowheads indicate two processes related to oogenesis. (D) Abundance of small RNAs ranging from 24 to 30 nt. Arrows indicate the decrease in piRNA abundance. (E) Fold changes of BmAsh2, BmSETD2 and BmEggless in Δsiwi and Δago3 females normalized to WT. Asterisks stand for significance with p<0.05. (F) Relative transcript abundance of BmSiwi in Δash2 ovaries. The silkworm ribosome protein 49 (Bmrp49) ortholog was used as the internal reference gene in (E) and (F). Three individual replicates were used for qRT-PCR, and the error bars represent the mean ± S.E.M. (TIF) [file pgen.1007245.s005.tif]

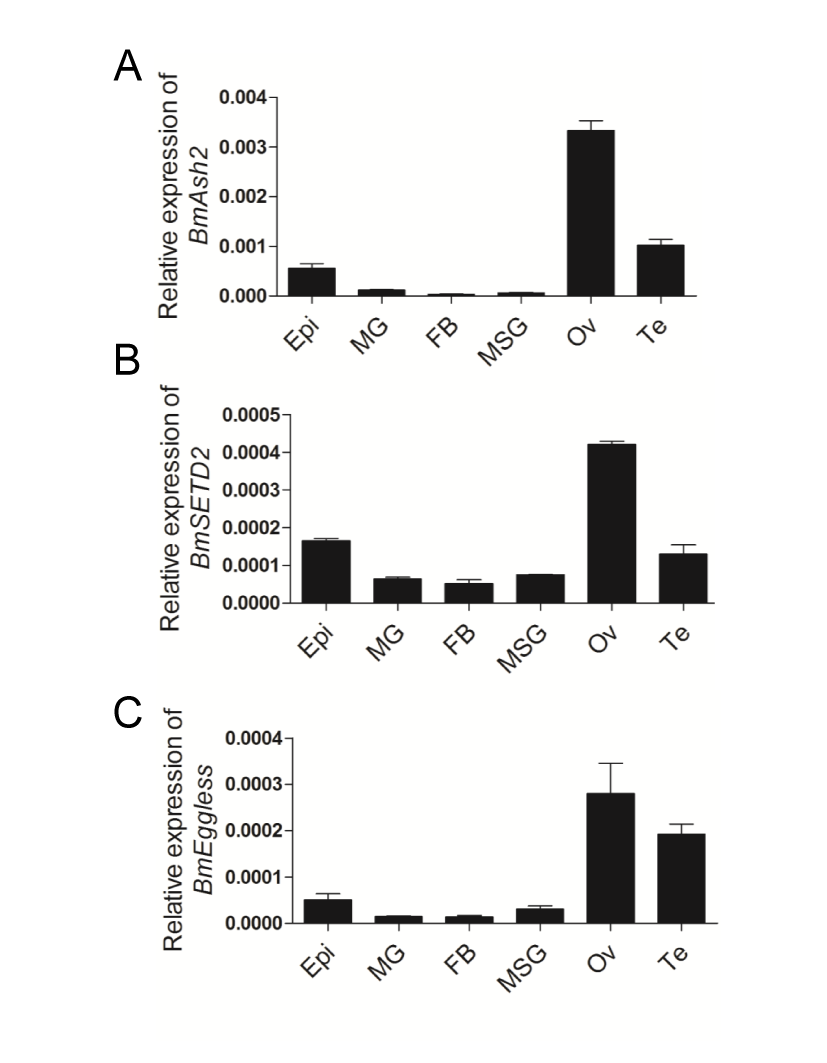

Supplement: S6 Fig — Six major tissues, including Epi, MG, FB, MSG, Ov and Te, were sampled from W larvae and used for investigation. Three individuals were used for qRT-PCR. The error bars represent the mean ± S.E.M. (TIF) [file pgen.1007245.s006.tif]

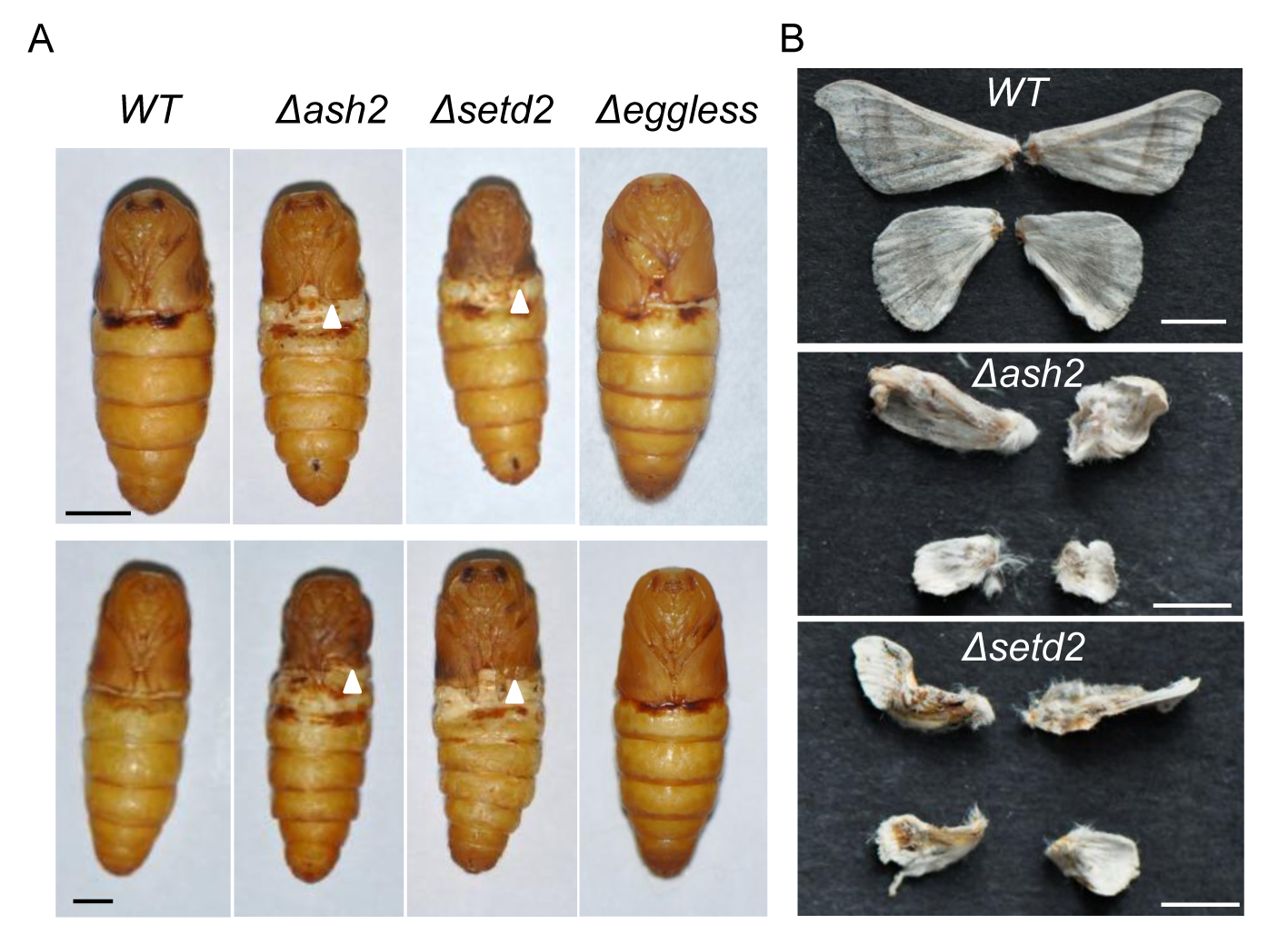

Supplement: S7 Fig — (A) Female (upper) and male (lower) pupae at day 5 after puparium. White arrowheads indicate abnormal wing discs in pupae of Δash2 and Δsetd2 animals. (B) Abnormal wings from WT adult, Δash2 and Δsetd2 day nine pupae. The upper are fore wings and lower are hind wings. Scale bars represent 0.5 cm. (TIF) [file pgen.1007245.s007.tif]
