# Supplementary material for: Bombyx mori histone methyltransferase BmAsh2 is essential for silkworm piRNA-mediated sex determination
Source: PLoS Genet. 2018 Feb 23;14(2):e1007245. doi: 10.1371/journal.pgen.1007245 (PMC5841826; doi:10.1371/journal.pgen.1007245)
Supplement: S1 Table — (DOCX) [file pgen.1007245.s008.docx]

**S1 Table. Primers used in this work.**

| **mRNA qRT-PCR primers** | | |
| --- | --- | --- |
| Targeting name | Forward primer (5’- 3’) | Reverse primer (5’-3’) |
| *BmRTAsh2* | GCTATGACCACAATGCCCAG | TGCCGGCTAGTGGAATTGTA |
| *BmRTEggless* | GACGAGGGAGCCAAGTTACT | CAGCATCTGGTCGATCTCCT |
| *BmRTSETD2* | TGGTGTACTGATGCTCCGTT | CAAACCGGTGGCTTCATCAA |
| *BmRTMasc* | GCTAAAATTGCTGGGATTGCTA | CAGCAATCGGAATTTTCTTCTG |
| **piRNA qRT-PCR primers** | | |
| *piRNA RTR* |  | CTCAACTGGTGTCGTGGAGTCGG |
| Targeting name | Stem-loop transcriptional primer | qRT-PCR forward primer |
| *U6* | CTCAACTGGTGTCGTGGAGTCGGCAATTCAGTTGAGACGATTTTGCGT | ACACTCCAGCTGGGCTGCGCAAGGATGACACG |
| *Fem* | CTCAACTGGTGTCGTGGAGTCGGCAATTCAGTTGAGATGACTTTAT | ACACTCCAGCTGGGTACCTCTTTTTGTCAATTC |
| *Masc* | CTCAACTGGTGTCGTGGAGTCGGCAATTCAGTTGAGTCTGATTAGCTTCA | ACACTCCAGCTGGGAAAAGAGGTAACAAT |
| *Judo1* | CTCAACTGGTGTCGTGGAGTCGGCAATTCAGTTGAGCATGCTACGT | ACACTCCAGCTGGGTAGGTCATGACGTCTT |
| *Judo2* | CTCAACTGGTGTCGTGGAGTCGGCAATTCAGTTGAGGCAAGCGCTA | ACACTCCAGCTGGGTTGTTTACAAATCGTTCA |
| *Suzuka* | CTCAACTGGTGTCGTGGAGTCGGCAATTCAGTTGAGCACCTATCGG | ACACTCCAGCTGGGAGGTCTTTAGTCATC |
| *Inoki* | CTCAACTGGTGTCGTGGAGTCGGCAATTCAGTTGAGTGTTAGCGTC | ACACTCCAGCTGGGAGTACTTATCACGCACT |
